# Comparative Evaluation of Antimicrobial Efficacy of MTAD, 3% NaOCI and Propolis Against *E Faecalis*

**DOI:** 10.5005/jp-journals-10005-1049

**Published:** 2010-04-15

**Authors:** Asha Nara, Dhanu, Prakash Chandra, Latha Anandakrishna, Dhananjaya

**Affiliations:** 1 Postgraduate Student, Department of Pedodontics and Preventive Dentistry, MS Ramiah Dental College and Hospital Bengaluru, Karnataka, India; 2 Professor, Department of Pedodontics and Preventive Dentistry, MS Ramiah Dental College and Hospital Bengaluru, Karnataka, India; 3 Professor and Head, Department of Pedodontics and Preventive Dentistry, MS Ramiah Dental College and Hospital Bengaluru, Karnataka, India; 4 Professor, Department of Pedodontics and Preventive Dentistry, MS Ramiah Dental College and Hospital Bengaluru, Karnataka, India; 5 Reader, Department of Pedodontics and Preventive Dentistry, MS Ramiah Dental College and Hospital Bengaluru, Karnataka, India

**Keywords:** BHI broth, *Enterococcus faecalis*, MTAD, 3% NaOCl, propolis.

## Abstract

**Aim:**

The present study sought to compare the antimicrobial efficacy of 3% NaOCl, Biopure MTAD (Tulsa Dentsply, Tulsa, OK) and Brazilian ethanolic extract of propolis (EEP) against *Enterococcus faecalis (E. faecalis).*

**Methodology:**

The study utilized 55 extracted human permanent teeth with single root canal. The samples were decoronated, instrumented and sterilized. The teeth were infected with *E faecalis* for 48 hours. The teeth were divided randomly into 3 groups according to the irrigants used and kept in contact with the respective irrigant for 5 minutes. All the samples were incubated in brain heart infusion (BHI) broth for 96 hours. Disinfection of the samples was determined based on presence or absence of turbidity in the BHI broth 96 hours later. Statistical analysis was done using Chi-square test.

**Results:**

All the samples treated with MTAD showed complete absence of turbidity, while all the 15 teeth treated with propolis showed presence of turbidity, 8 out of 15 teeth treated with NaOCl showed presence of turbidity. Statistical analysis of the data using chi-square test showed significant difference between the groups (P < 0.05).

**Conclusion:**

The study concluded that MTAD was more effective than 3% NaOCl and propolis against *E. faecalis.*

## INTRODUCTION

Microorganisms play fundamental role in etiology of pulp and periradicular lesions.^1^ Successful root canal therapy relies on triad of instrumentation, disinfection and obturation.^2^ Disinfection of the root canal is major determinant in the healing of periapical tissues.^1^ Though the chemomechanical preparation and use of antimicrobials are effective in reducing the bacterial load, some bacteria can still persist.^3^ *Enterococcus faecalis* is one among the facultative organism which is persistently found in root canal failures,^4^ and is resistant to various intracanal medicaments.^5^ The microorganisms found in the root canals of deciduous teeth are similar to those in the root canals of permanent teeth.^6,7^

Though sodium hypochlorite (NaOCl) is a commonly used root canal irrigant, it has an unpleasant odor and taste; it does not consistently disinfect the root canal system^8^ and is toxic when extruded into the periradicular tissues.^9^ Because of these limitations, a search for better root canal irrigant continues.

Torbinejad in 2003 introduced a new irrigant, Biopure MTAD (Tulsa Dentsply, Tulsa, OK) a mixture of Doxycyc-line, citric acid, and Tween-80, which is capable of safely removing the smear layer^10^ and eliminating *E. faecalis.^11^*

Propolis, a resinous bee-hive product, is a potent antimicrobial, antioxidant and anti-inflammatory agent. The pharmacologically active constituents in propolis are flavonoids, phenolics and aromatics. Propolis has been used in dentistry as pulp capping agent,^12^ as storage media for avulsed teeth,^13^ for prevention of caries^14^ and dentine hypersensitivity.^15^ But its use as a root canal irrigant has not been evaluated yet.

So the present study sought to compare the antimicrobial efficacy of 3% NaOCl, MTAD and propolis against *E. faecalis.*

## MATERIAL AND METHODS

The methodology used was adapted from the research study protocol of Shabahang and Torbinejad with some modifica-tions.^16^ Fifty-five extracted single rooted human permanent teeth were collected and stored in 10% formalin. Each tooth was radiographed with digital radiography to confirm the presence of patent canal. The teeth were decoronated and instrumented 0.5 mm short of apex up to size 40 k-type file maintaining 10 mm of working length. One ml of NaOCl was used for cleaning and shaping. One ml of 17% of EDTA was used to remove the smear layer. Later all the roots were autoclaved. Five autoclaved samples were transferred to sterile broth to serve as negative control.

A 24 hours culture of *E. faecalis* (ATCC 4082) was grown in BHI broth with concentration of1 × 10^8^ cells/ml. Each root canal was inoculated with 1 μl of *E. faecalis* suspension using a sterile 1mL tuberculin syringe and incubated at 37°C for 48 hours. Five infected samples were kept as positive control. The infection of the specimen was confirmed by sampling the culture on McConkey’s agar. Growth was determined by visualization of individual white pin point colonies on agar plates. The rest of the contaminated samples were then divided into 3 experimental groups according to the irrigant used.

*Group I:* 15 samples were irrigated with 2 ml of 3% NaOCl and kept in the canal for 5 minutes.

*Group II:* 15 samples were irrigated with 2 ml of Biopure MTAD (Tulsa Dentsply Tulsa, OK) and kept in the canal for 5 minutes. (MTAD prepared according to manufacturer’s instructions).

*Group III:* 15 samples were irrigated with 2 ml of Propolis and kept in the canal for 5 minutes (Flow chart 1).

All the samples were then washed with distilled water to prevent potential carry-over of the irrigants, transferred into another tubes containing 2 ml of BHI broth and aseptically cultured in an incubator at 37°C for 96 hours. After 96 hours, two observers blinded as to the irrigant examined the tubes for the presence of turbidity. The numbers of positive and negative samples were recorded.

The chi-square test was used for statistical analysis to evaluate the differences between the groups.

## RESULTS

All of the positive control samples caused turbidity in the tubes, whereas none of the negative controls showed turbidity. Data obtained for each irrigation regimen are presented in Table 1. Eight out of fifteen samples treated with 3% NaOCl remained contaminated as evidenced by turbidity of the BHI broth after 96 hours. While all the 15 samples which received irrigation with Propolis showed presence of turbidity, none of the samples treated with MTAD showed turbidity. There was statistical significant difference between samples treated with 3% NaOCl, MTAD and Propolis (p < 0.05).

**Table 1:** **Table 1:** Comparision of antimicrobial effect of 3% NaOCl, MTAD and propolis

*Groups*		*Sample size*		*Growth*		*No growth*	
Negative control		5		0		5	
Positive control		5		5		0	
3% NaOCl		15		8		7	
MTAD		15		0		15	
Propolis		15		15		0	

## DISCUSSION

One of the main aims of root canal treatment is to eliminate the bacteria, their byproducts and the substrate from the root canal system.^17^ The use of irrigation solution in this process is essential to ensure bacterial elimination and eradication of organic tissue remnants.^18^ Maximum antibacterial effect, maximum tissue dissolving effect on the necrotic tissues and the least toxic effect on the peripheral tissues are some important features of an ideal root canal irrigant.^19^ The complex morphology and the irregularity of the root canals of primary teeth negatively affect the success of chemo-mechanical endodontic treatment.^20^ Sodium hypochlorite is, till date, the most commonly employed root canal irrigant.^21^ The antimicrobial activity of NaOCl is by the release of hypochlorous acid (HOCl) and its oxidative action on sulfhydryl groups of bacterial enzymes thereby disrupting the metabolism of the microorganism.^21^ Although it is an effective antibacterial agent, NaOCl is toxic when extruded to the periradicular tissues.^19^ In root canal treatment of primary teeth, NaOCl can damage permanent tooth follicles, peripheral tissues and oral mucosa.

Therefore, research for new irrigants continues. MTAD, after its introduction in 2003 was subjected to various test procedures to evaluate its efficacy and was compared with various commonly used irrigants. The superior antimicrobial activity of MTAD over 3% NaOCl seen in this study are in agreement with the findings of Shabahang and Torabinejad’s study.^11,16^

In present study the smear layer was removed before contaminating the teeth with *E. faecalis* to allow penetration of bacteria into the tubules. The lack of turbidity of the BHI by negative control group demonstrated that the sterilization procedure utilized was effective. The results from the samples in the positive group confirmed the presence of *E. faecalis* within the root canal systems. The roots were immersed in culture media for 96 hours so that if bacteria were present in the dentinal tubules, their movement into the main canal would result in turbidity of the media. The samples treated with MTAD showed absence of turbidity proving efficacy of MTAD deep into the canals. This effect is related to presence of detergent in it.

**Flow chart 1:**
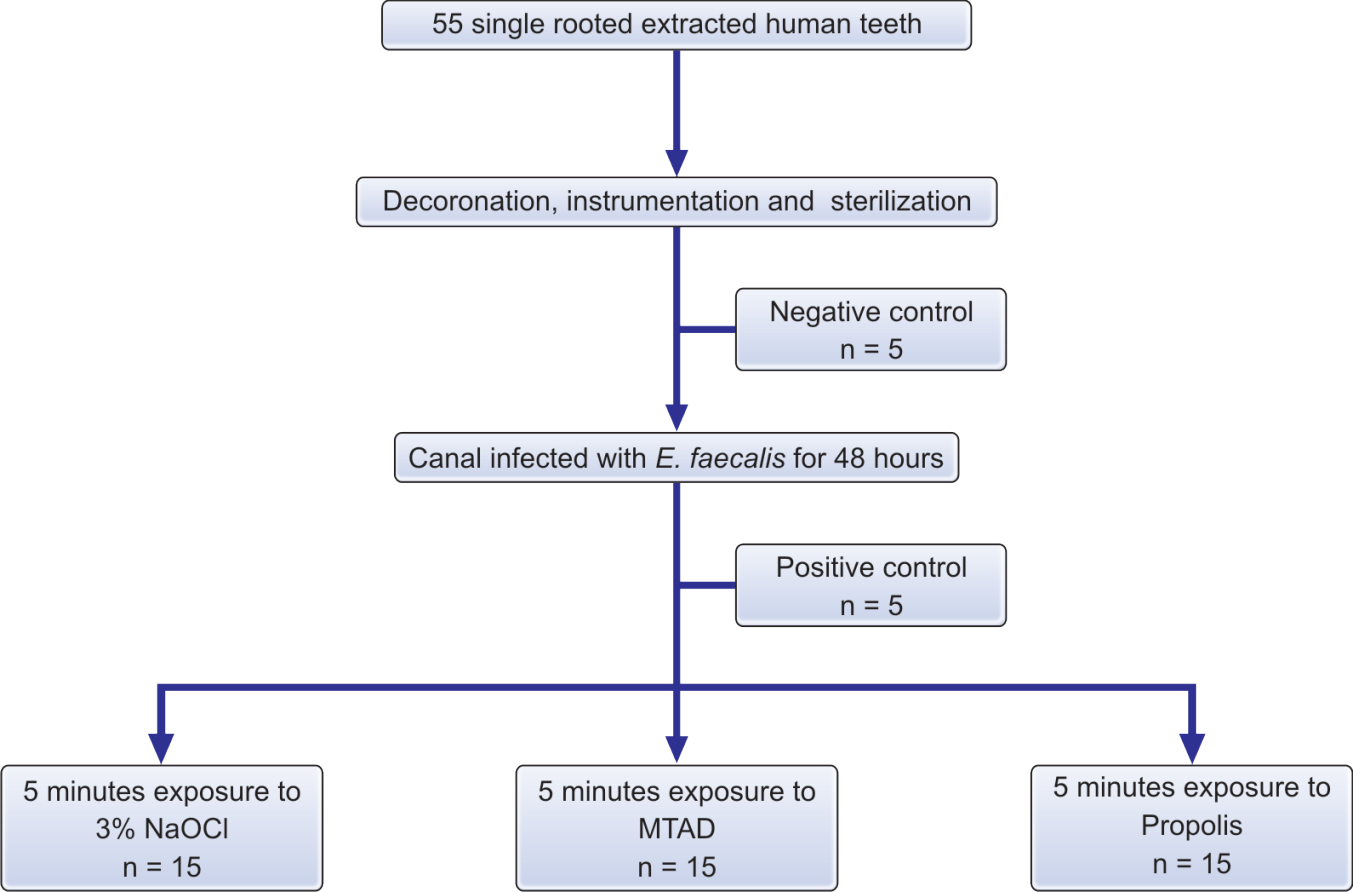
Distribution of samples among the treatment groups

The organism *E. faecalis* was selected in this study because it is most commonly isolated in endodontic retreatment of apical periodontitis^4^ and has been identified to be resistant to currently used chemicals such as sodium hypochlorite,^21^ potassium iodide^22^ or calcium hydroxide^23^ and has been found to survive as a monoinfection in root canals.^24^

Previous *in vitro* studies have shown a high level of susceptibility of *E. faecalis* to MTAD, even when this solution was diluted 200 times, whereas NaOCl loses its antibacterial activity against the same isolate beyond 32 times dilution.^11^ MTAD removes the smear layer with significantly less erosion of the dentinal tubules compared to EDTA.^10^ Also when MTAD was evaluated for biocompatibility, it was found to be less cytotoxic than Eugenol, 3% H_2_O_2_, Ca (OH)_2_ paste, 5.25% NaOCl, 0.12% Chlorhexidine gluconate, and EDTA and more cytotoxic than 2.63%, 1.31% and 0.66% NaOCl.^25^ The antimicrobial efficacy of MTAD is because of anticol-lagenase activity of Doxycycline, its low pH, ability to be released gradually over time,^10^ and its action is facilitated by citric acid which removes the organic and inorganic substances. Tween-80 reduces surface tension on the dentinal tubules and allows deeper penetration of Doxycycline into the tubules.

One of the significant features of MTAD is its capacity to kill *E. faecalis* after a mere exposure of 5 minutes making it useful in the clinical situation. However this effect was not seen with NaOCl.^26^ Newberry et al showed that MTAD inhibited most strains of *E. faecalis* growth when diluted 1:8192 times and killed most strains of *E.faecalis* when diluted 1:512 times.^27^ Thus MTAD has properties of an ideal root canal irrigant.

The results of the present study are in accordance with the findings of Portenier et al,^28^ Ghoddusi et al,^29^ and Davis et al,^30^ while studies done by Dunavant et al^31^, Baumgartner et al,^32^ Krause et al^33^ are in disagreement with these results. The disparity in the results may be caused by differences in methodology and variance in strains tested. Failure to follow manufacture’s recommendations may affect the results of the study. Torabinejad’s group recommended the use of 1.3% NaOCl for 15 to 20 minutes before the final rinse with MTAD.^26^ Some of these studies^31,33^ did not use NaOCl at all or used it for a shorter period than recommended. Use of MTAD in primary teeth is however limited due to chance of discoloration of permanent tooth buds present below the primary teeth. However its use in permanent teeth in younger age may not be controversial.

Natural products have been used in dental and medical practices for thousands of years and have become even more popular today. Propolis has gained increased interest due to its antimicrobial activity against a wide range of pathogenic organisms.^34^ Propolis is collected from various plant sources by honeybees and its chemical composition varies due to climate, season and location. The main chemical classes present in propolis are flavonoids, phenolics and aromatic compounds. Flavonoids are well known compounds that have antioxidants, antimicrobial, antifungal, antiviral and anti-inflammatory properties. Brazilian propolis is characterized by a very low concentration of flavonoids and esters of phenolic acids, but it has a high concentration of dihy-drocinnamic acid, prenylated acetophenones and specific terpenoids, which have antimicrobial activity.^35^

Though in the present study Propolis found to be less efficacious than NaOCl and MTAD, Al-Qathami and Al-Madi found that propolis was as equally effective as NaOCl when used as an antimicrobial irrigant on extracted human teeth.^36^ Koo et al tested the antimicrobial efficacy of EEP, using agar diffusion method and observed that it significantly inhibited all of the microorganisms tested, including facultative and strict anerobic species.^37^ Oncag et al observed that Propolis has good antibacterial activity against *E. faecalis* in the root canal of extracted teeth suggesting that it can be used as an alternative intracanal medicament.^38^ Gafar et al used intracanal dressings of EEP in infected teeth and showed that it gave better results than camphorated paramonochlo-rophenol.^39^ When utilized as a pulp-capping agent, EEP induced hard tissue bridge formations in the same way as those induced by calcium hydroxide.^40^ Al Shaher et al using pulp and periodontal fibroblast cell culture observed that propolis exerts minimal toxicity on either type of cell.^41^

Propolis has shown biocompability and proven its antibacterial efficacy in the pulp - periapical infections. It is important to remember that the flavonoid composition of propolis is qualitatively and quantitavely different depending on the region where propolis is collected. So to include in the arsenal of intracanal substances further research such as *in vivo* and *in vitro* studies are required for better understanding of action of propolis as an intracanal irrigant.

To conclude, in the present study biopure MTAD has been shown to be a better irrigant when compared to 3% NaOCl and propolis. Further *in vitro* and *in vivo* studies are essential to validate the use of propolis as an irrigant against *E. faecalis.*
